# Travel-associated Rabies in Austrian Man

**DOI:** 10.3201/eid1105.041289

**Published:** 2005-05

**Authors:** Robert Krause, Zoltán Bagó, Sandra Revilla-Fernández, Angelika Loitsch, Franz Allerberger, Peter Kaufmann, Karl-Heinz Smolle, Gernot Brunner, Guenter J. Krejs

**Affiliations:** * Medical University of Graz, Graz, Austria;; † Institute for Veterinary Disease Control, Mödling, Austria

**Keywords:** Rabies, dog, Morocco, Austria

## Abstract

Rabies developed in an Austrian man after he was bitten by a dog in Agadir, Morocco. Diagnosis was confirmed by reverse transcription–polymerase chain reaction and immunohistochemistry. The patient's girlfriend was bitten by the same dog, but she did not become ill.

Rabies is an acute, progressive, fatal encephalomyelitis that can be prevented by vaccination. It is almost always transmitted by the bite of an infected animal and is still a public health problem in many countries in Africa and Asia. Travelers with extensive unprotected outdoor exposure, such as camping, in areas where rabies is endemic are also at high risk (1). We report a case of rabies associated with a dog bite in Agadir, Morocco, in an Austrian man.

## The Case

Two Austrian tourists (a 23-year-old man and a 21-year-old woman) traveled to Morocco in May 2004. At the end of July, they were staying in Agadir, Morocco, where they played with puppies on the beach. One puppy was aggressive and bit the woman on the third finger of her right hand. While attempting to assist her, the man was also bitten on the right hand and leg. The wounds healed without further treatment, and the young people did not seek medical assistance. Three days after the bite, the dog died and was buried by the tourists. Four weeks later, the man became ill with a temperature up to 39°C, malaise, pain in the right arm, headache, feeling of extreme dryness in the mouth, and difficulty swallowing. Two days later, he was admitted to a hospital in the Spanish enclave of Ceuta, where he additionally showed hydrophobia, aerophobia, agitation, and increased salivation. Subsequently, hyperventilation, decreased blood pressure of 85/45 mm Hg, heart rate of 150 beats/min, markedly increased salivation, and a generalized tremor developed.

The patient was transferred to the intensive care unit, intubated, mechanically ventilated, and treated with intravenous fluids, dopamine, ampicillin, cefotaxime, and vancomycin because of hypotension and pneumonia. The patient's history suggested rabies, and he and his girlfriend (who was healthy) received rabies vaccination and human rabies immunoglobulin (20 IU/kg intramuscularly in the gluteal area). Three days after admission to the Spanish hospital, the patient was transferred by air ambulance to the intensive care unit at the Medical University of Graz, Austria. Critical care management, treatment of pneumonia with vancomycin and cefotaxime, and administration of rabies vaccine (Rabipur, Chiron, Marburg, Germany), following the 0-, 3-, 7-, 14-, 28-day regimen, were continued. In addition to remifentanil and midazolam, ketamine was administrated. According to recent recommendations, we did not use ribavirin or interferon-α (2). Skin biopsy specimens from the neck and cerebrospinal fluid, pharyngeal swab, nasal swab, and serum specimens were sent to the National Reference Laboratory for Rabies (Austrian Agency for Health and Food Safety, Institute for Veterinary Disease Control, Mödling, Austria) and to the rabies laboratory at the Centers for Disease Control and Prevention (CDC), Atlanta, Georgia, USA.

**Figure 1:**
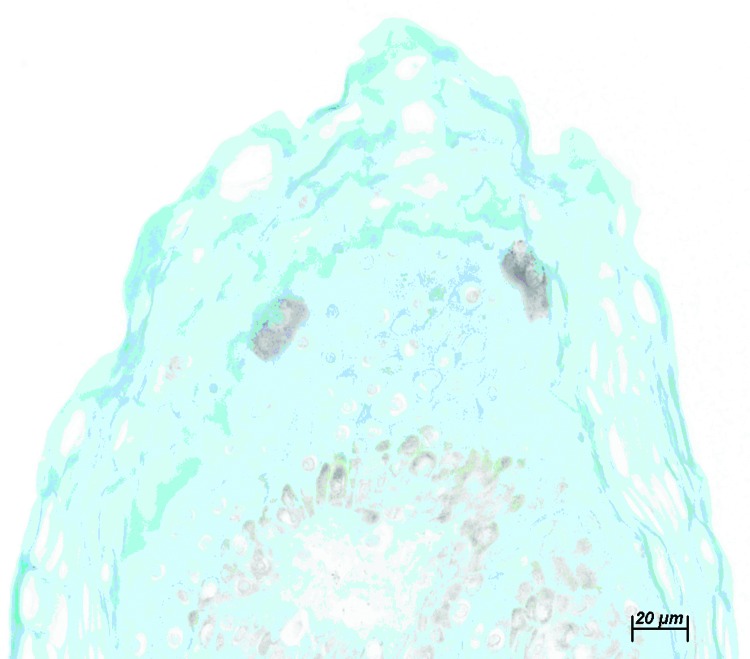
Immunohistochemical stain of neck biopsy specimen. Note positive cells with small intracytoplasmic granules at the border of stratum granulosum and stratum corneum of the epidermis. Bar = 20 μm.

**Figure 2:**
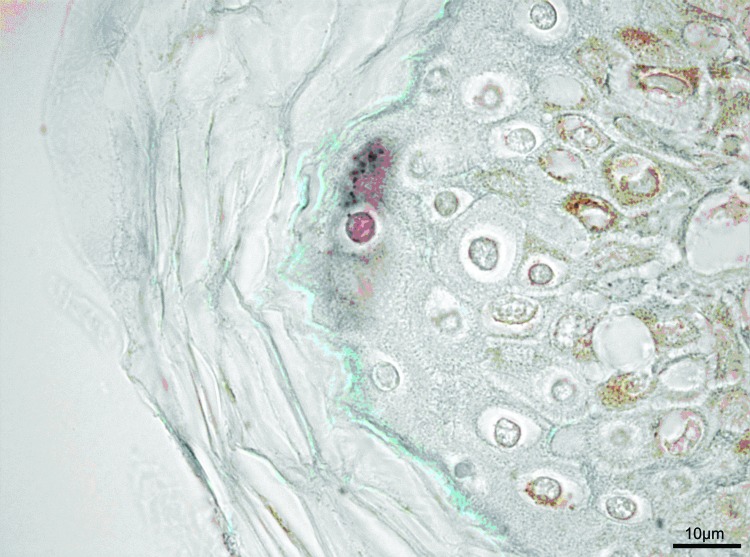
Immunohistochemical stain of neck biopsy specimen. Note the positive cell in the center with small intracytoplasmic granules at the border of stratum granulosum and stratum corneum of the epidermis. On the right side, melanin-rich epidermal cells are seen. Bar = 10 μm.

Skin and pharyngeal swab were positive by reverse transcription–polymerase chain reaction, showing 965-bp (Mödling) and 300-bp (CDC) DNA amplicons of the rabies virus nucleoprotein gene closely related to other strains from Morocco (GenBank accession nos. U22852, U22642, AY 062090, and U22631) (3). In addition, fluorescence antibody test results and immunohistochemical investigation of the neck biopsy specimen were positive (Figures 1 and Figure 2). Similar test results were also obtained from the Spanish National Center of Microbiology, Instituto de Salud Carlos III, Madrid, which received the patient's samples from Ceuta. Rabies serum antibody tests performed 4 and 20 days after onset of symptoms showed 0.38 U/mL and 52.09 U/mL, respectively. The neurologic symptoms of rabies worsened in the intensive care unit in Graz until pupils did not react to light. Twenty days after admission, 2 different electroencephalograms showed no brain activity, and life support was discontinued. The patient died 21 days after admission to our hospital and 27 days after onset of rabies symptoms.

Since the female Austrian tourist was bitten by the same rabid dog, she was also admitted to the Medical University of Graz and received a thorough examination and psychological support. No abnormalities were found. Rabies vaccination, which had been started in Ceuta, was continued, and she was released from the hospital in healthy condition. One week after complete vaccination, her antibody titer was 118.53 U/mL. She remained healthy 4 months after the bite and is currently being monitored.

## Conclusions

Our patient acquired rabies from a dog bite in July 2004 in Agadir, Morocco. On September 1, 2004, the World Health Organization announced that a rabid dog had been illegally imported into France from Agadir in July 2004. The dog was aggressive and bit several persons who were later contacted by French health authorities to assess their risk for infection and provide professional help, but no case of rabies associated with this dog has been reported to date (4,5). Our patient's dog bite–associated case of rabies underlines the potential risk for infection by dogs from Agadir and other rabies-endemic areas (4,5). This case of rabies is the first to be diagnosed and treated in Austria since 1979. Because of official rabies vaccination policy, Austrian domestic and wild animals have been free of rabies since 1995, with rare exceptions. Rabid foxes occasionally immigrate to Austria, as was the case in 2002 on the Carinthian-Slovenian border, and transmit rabies to other animals (foxes, deer, dogs, cats, badgers) (6–8). The occasional reappearance of rabies in Austria usually leads to vaccination campaigns in relevant areas; these campaigns, in combination with routine animal vaccination, prevent the dissemination of rabies (6). The general public in Austria thus does not consider rabies to be a serious problem, and recommendations for vaccination for travelers to rabies-endemic countries are sometimes ignored.

Although preexposure rabies prophylaxis is recommended for travelers to Morocco (9), our patient and his girlfriend did not receive vaccination before their trip. They did also not seek medical assistance or receive postexposure prophylaxis immediately after the bites because they did not understand the enormous potential risk from a dog bite in a country where rabies is endemic. After the patient showed symptoms indicative of rabies 1 month later, he and his girlfriend were admitted to a hospital and received passive and active postexposure prophylaxis. Whereas the lethal course of the disease could not be prevented in the man, the woman remained healthy, and high levels of rabies antibodies developed. However, in 5% of rabies cases the incubation period exceeds 1 year (10). Since this young woman was bitten by the same dog that transmitted rabies to our patient, she is still being followed. Our case of travel-associated rabies in an Austrian man underscores the importance of adhering to vaccination recommendations and the need for providing detailed information to travelers.

Recently, authorities have recommended that molecular diagnostic methods with samples from several sources be performed and repeated until the diagnosis of rabies is established. However, molecular diagnostic methods, although useful and sensitive, may not always give positive results for patients with rabies (11). This lack of sensitivity may be due to the intermittency of virus shedding, the timing of sample collection, and the type of specimens collected. For this reason and in concordance with CDC recommendations, we suggest that immunohistochemical investigation of skin biopsy specimens should be performed for antemortem diagnosis of rabies, as we did with our patient (12).
